# A Solid Pseudopapillary Tumour Arising from Mesocolon without Ectopic Pancreas

**DOI:** 10.1155/2010/206186

**Published:** 2011-01-20

**Authors:** Mesut Tez, Necdet Özalp, Barış Zülfikaroğlu, Mahmut Koç

**Affiliations:** Fifth Department of General Surgery, Ankara Numune Training and Research Hospital, Ankara 06100, Turkey

## Abstract

A solid pseudopapillary tumour (SPT) is an uncommon pancreatic tumour. Very rarely it has also been described outside the pancreas, usually arising from heterotopic pancreatic tissue. In this paper, we described a case arising from the transverse mesocolon without heterotopic pancreatic tissue in an 18-year-old girl.

## 1. Introduction

Solid pseudopapillary tumour (SPT) of the pancreas is one of the rare found primary tumors of the pancreas, making up approximately 0.17–2.7% of cases [1]. There have been controversies regarding the terminology and according to its macroscopic and microscopic pathological characteristics over the years. It has been given multiple descriptive names such as papillary-cystic tumour, solid cystic tumor, papillary epithelial neoplasm, solid, and papillary neoplasm, papillary tumour of the pancreas, or Frantz's tumor [1] until in 1996 the WHO pancreatic tumour working group recommended the use of the term solid pseudopapillary neoplasm [2]. One of its characteristics is that this tumour seems to preferentially affect mainly young women usually in the second or third decade of life [3]. Furthermore, SPT rarely develops outside the pancreas. [4, 5]. Extrapancreatic SPT cases in the retroperitoneum, liver, omentum, and mesocolon have been published. Some of them were considered to arise from an ectopic pancreas [4–6].

To the best of our knowledge, this is the first SPT originating from mesocolon without ectopic pancreas tissue.

## 2. Case Report

**Figure 1:**
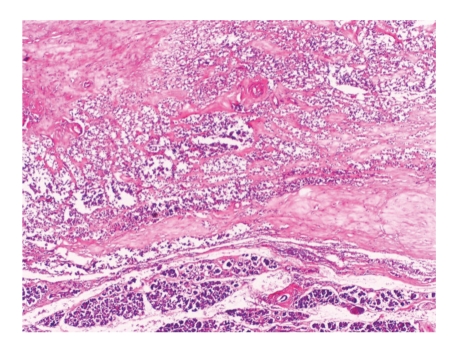
Small cells with uniform spherical nuclei with narrow eosinophilic cytoplasm and tumoral cells forming glandular structures.

**Figure 2:**
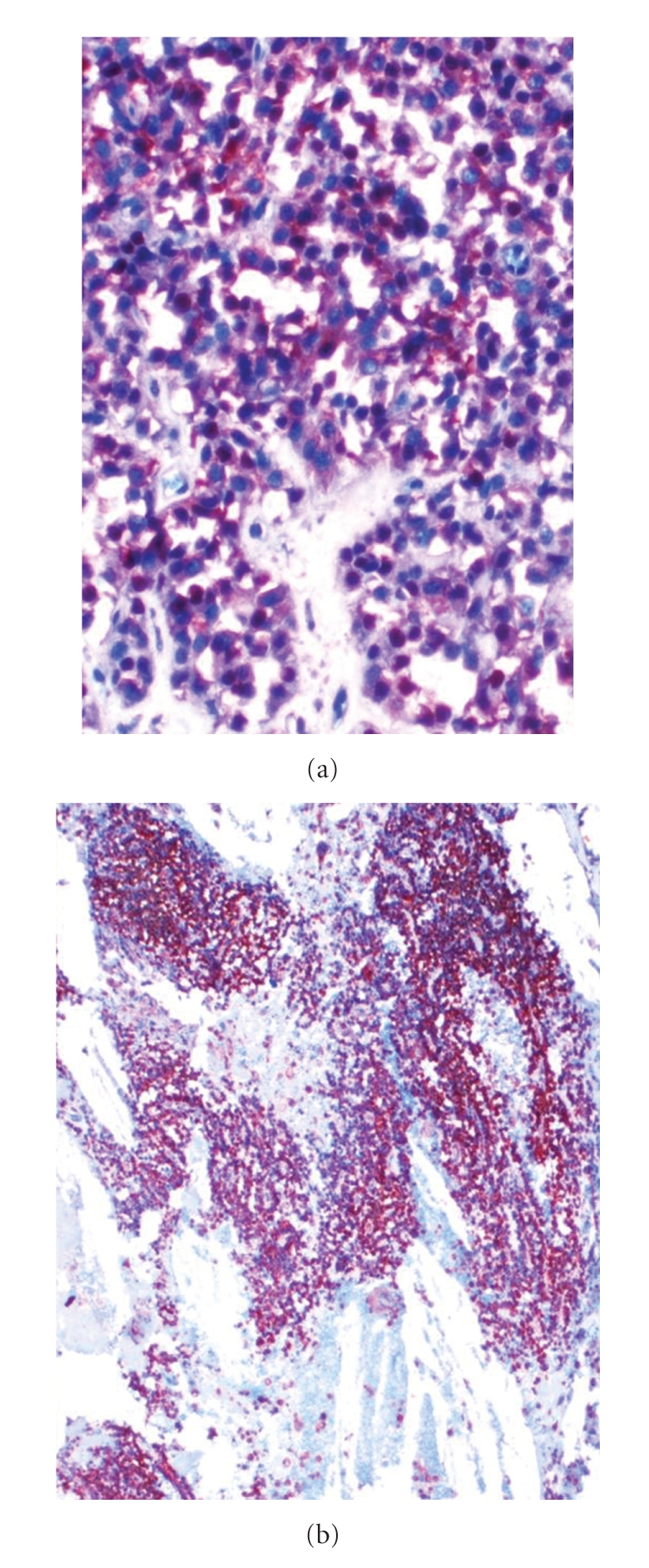
Tumoral cells were stained remarkably with progesterone receptor antibody (a) and vimentin (b).

An 18-year-old woman complaining of abdominal pain, nausea, and vomiting for one month was admitted to our hospital for further evaluation. She had no history of abdominal trauma or surgery, drug usage or smoking. Physical examination was normal. Laboratory findings only revealed anemia (hemoglobin: 9.2 g/dL). Tumour markers (alpha-fetoprotein, carcinoembryonic antigen, CA-125, CA15-3, CA19-9, and CA72-4) of serum and other biochemical analyses were within normal limits. Abdominal Ultrasonography (USG) showed an encapsulated solid mass of 5 × 5.5 cm in diameter located on the subhepatic region displacing the second part of the duodenum laterally, and the computer tomography confirmed a well-encapsulated mass of 48 × 51 mm diameter located in the mesenteric region neighboring the superior mesenteric artery and vein, with no distinct separation from the head of the pancreas. The patient then underwent surgery, and exploration revealed an encapsulated mass of 5 × 5 cm in diameter that was adherent to the mesentery of the transverse colon. The mass was dissected from the mesentery and duodenum, preserving the pancreas. The tumor was not infiltrating the pancreatic tissue. No vascular invasion or lymph node metastasis was detected. Pathologic examination of biopsy material revealed small cells with uniform spherical nuclei with narrow eosinophilic cytoplasm and tumoral cells forming glandular structures (Figure 1). Neuron-specific enolase (NSE), chromogranin, CD10, CD99, CD68, LCA, Calcitonin, CEA, EMA, HMW CK, LMW CK, CK 7, Ki 67, and synaptophysin antibody staining were negative; however, the tumoral cells were stained remarkably with progesterone receptor antibody and vimentin (Figures 2(a) and 2(b)). In light of these findings, primary pathological diagnosis was SPT. No definitive structures suggesting ectopic pancreas were observed.

Postoperative recovery was uneventful, and the patient was discharged with complete recovery.

## 3. Discussion

SPT is a rare disease with a reported incidence of 0.13% to 2.7% of all pancreatic tumors [3], and cases often have been misunderstood. The histogenesis is still poorly understood, and the molecular pathological data are limited [5].

The SPT has usually occurred in young women during the second to fourth decades of life. Regardless of the location, however, pain was the most common initial symptom [7]. Abdominal discomfort is the prevailing symptom associated in some cases with a palpable mass, anorexia, and weight loss [7].

It is uncommon for an SPT to develop outside the pancreas [4–7]. The most common extrapancreatic sites are mesocolon, liver, retroperitoneum, or greater omentum [4–7].

Only four SPT, developed from mesocolon, have been described previously (Table 1). The presence of ectopic pancreatic tissue is seen in all of these published cases [5, 6, 8, 9].

Ishikawa et al. [6] described the first SPT case arising from an ectopic pancreas in the mesocolon. Patient was 13-year-old girl, and 8-cm, well-encapsulated, and partly calcified tumor which protruded from the mesocolon was resected surgically. At the base of this tumor, small pancreatic tissues (islet, acinar, and ductular cells) were detected in the mesocolic tissue [6]. These four tumours (Table 1) tended to grow to a large size (60–210 mm), usually occurred in young female patients (only one was in a man), and produced similar clinical signs. In all four published cases the tumour was found to be separate from the main pancreas at surgery.

A case of SPT arising in the omentum without pancreatic tissue was published previously [4]. Kosmahl et al. [10] mentioned that the occurrence of a few SPTs in the retroperitoneal space outside the pancreas can be related to the localization of the genital ridge during embryogenesis [1] and speculated that SPT might originate from genital ridge-related cells that were incorporated into the pancreas during organogenesis. This theory might suggest one explanation for the occurrence of SPT in the mesocolon in the presented case.

SPT can be readily diagnosed by routine histologic examination, but accuracy of diagnosis may be improved with the help of immunohistochemical staining because such tumours are typically negative for cytokeratin, pancreatic enzyme markers, and endocrine markers but positive for vimentin, CD 10, CD 56, and alpha-antitrypsin [3–7, 9, 10].

Surgery is the only curative treatment for extra pancreatic SPT.

## 4. Conclusion

We encountered an extremely rare case of SPT arising in the mesocolon without ectopic pancreatic tissue. It is therefore doubtful that they originate from the pancreas.

## Figures and Tables

**Table 1:** Summary of the present and previously published cases.

Author	Sex/Age	Symptom	Size (mm)	Heterotopic pancreas	Procedure
Ishikawa et al. [6]	F/13	Mass	80	Yes	Resection
Tornóczky et al. [5]	F/15	Pain, mass	210	Yes	Resection with normal colon
Klöppel et al. [9]	M/25	None	80	Yes	Resection
Elorza Orúe et al. [8]	F/33		60	Yes	Resection
Present case	F/18	Pain	50	No	Resection
